# Metachronous Colon Metastasis to the Thyroid: A Case Report and Literature Review

**DOI:** 10.1155/2013/241678

**Published:** 2013-12-02

**Authors:** Dvir Froylich, Eitan Shiloni, David Hazzan

**Affiliations:** ^1^General Surgery B Department, Carmel Medical Center, 34362 Haifa, Israel; ^2^Minimally Invasive Surgery, Surgery B Department, Carmel Medical Center, 34362 Haifa, Israel

## Abstract

The thyroid gland is a known site for metastatic tumors from various primary sites. Thyroid metastases are not an exceptional finding at autopsy, and they are encountered in 2% to 9% of the patients with malignant neoplasm. The most frequent tumors to metastasize are breast, lung, melanoma, and kidney carcinomas. Despite the fact that it is one of the largest vascular organs in the body, clinical and surgical cases have given an incidence of 3% of secondary malignances of this organ. Metastatic colon carcinoma to the thyroid gland has been reported, and it is not as rare as one might think. We present a very unique case of colon carcinoma metastasis to the right thyroid lobe and lung five years after colon resection, with reoccurrence two years later in the contralateral thyroid lobe. The literature regarding colon cancer metastasizing to the thyroid gland was reviewed with an attempt to disclose features of this presentation regarding patient's prognosis.

## 1. Introduction

Microscopic metastasis to the thyroid gland is not rare, having been found in 4%–9% of autopsy studies [1]. The most frequent tumors to metastasize are breast, lung, melanoma, and kidney carcinomas [2]. However, gastrointestinal and particularly colonic metastases to the thyroid are extremely rare and usually associated with lung and liver metastasis [3]. A literature search identified 34 reported cases of colon carcinoma metastasis to the thyroid. 

We present a case of colon carcinoma metastasis to the right thyroid lobe and lung five years after colon resection, with recurrence two years later in the contralateral thyroid lobe. To the best of our knowledge, metachronous colon metastasis to the thyroid has never been reported previously. This unique case is presented and the published literature of colonic carcinoma metastasized to the thyroid is reviewed.

## 2. Case Presentation 

A 65-year-old female with hypothyroidism had a laparoscopic left colectomy due to sigmoid carcinoma (T3, N1, M0) in March 2005. Adjuvant chemoradiotherapy was administrated postoperatively and included FOLFIRI (5-FU, leucovorin and irinotecan) as well as pelvic radiation due to positive colonic margins. A CT scan performed four months after the surgery showed an enlarged right thyroid lobe with a nodule. Consequently, a PET CT was performed and revealed a pathological uptake in the right thyroid lobe. Fine needle aspiration (FNA) demonstrated normal cytology.

In December 2009, the patient's carcinoembryonic antigen (CEA) rose and a CT of the chest and abdomen demonstrated possible metastatic lesions in the right lung and pelvis. FNA of the lung showed adenocarcinoma originating in the colon.

In March 2010, the patient underwent a right upper and middle lobectomy followed by chest radiotherapy. Three months later, an anterior resection of the rectum was performed because of anastomotic recurrence and pelvic metastasis positioning on the sigmoid mesenterium. Xeloda (capecitabine) was given as adjuvant chemotherapy postoperatively.

A PET CT was performed in November 2010 and again demonstrated a pathological uptake in the right thyroid lobe. FNA revealed atypical cells without follicular or papillary differentiation. Right thyroid lobectomy was performed. The final pathological report suggested metastasis of colon carcinoma. FOLFOX adjuvant therapy was administered postoperatively.

In November 2011, an enlarged nodule was palpated on the patient's left thyroid lobe. A CT scan demonstrated a 2 cm nodule in the left lobe. The patient underwent complete thyroidectomy in June 2012. Final histological summation reported colonic metastasis to the left lobe of the thyroid.

## 3. Discussion

Colon adenocarcinoma metastasized to the thyroid is rare, usually detected years following colectomy, and most commonly is associated with metastasis to organs such as lungs and liver. There have been reports describing a thyroid mass as an initial presentation and thyroid metastasis without other organ involvement, but these reports are rare [5, 6]. Primary thyroid neoplasms are far more common than secondary lesions, but in the presence of other history of malignancy, the risk of metastasis is probably higher.

Cases that have been published in the literature are summarized in Table 1. We found 34 cases, two thirds being female. Patients' age ranged from 34 to 85. The metastasis origin was from the rectum (11 cases), sigmoid colon (9), right colon (5), and left colon (3). In 7 cases, the primary colonic location was not mentioned. The staging of the colon carcinoma was specified in only 20 reports, and at presentation there was stage III or IV in 75% of the patients. Metastasis to the thyroid was diagnosed 6 months to 8 years after colonic resection. However, in two cases, metastasis was diagnosed synchronously with the colon carcinoma. In 11 patients, a primary thyroid pathology was diagnosed in addition to the colon metastasis. 

In 29 cases, other organ involvement was noted. The lung was the most frequently involved organ in cases of widespread disease, leading to a poor prognosis of survival of a few months following diagnosis. However, some reports showed a more prolonged survival of 2 or 3 years at least, as in this present case. Those patients all had diffuse disease and were either managed with chemotherapy and thyroidectomy or radiation. In our case, signs of metastasis were demonstrated by PET-CT 2 years before diagnosis but failed to be proven by FNA. This questions FNA as a reliable diagnostic modality for metastasis to the thyroid. 

With regard to the primary tumor location, 19 patients had either a rectal or sigmoid adenocarcinoma. Metastasis to the thyroid could be via two routes: the portomesenteric or the caval. Some reports mentioned in Table 1 were about right sided colon carcinoma without liver involvement. This could be explained by drainage of colon metastasis to the inferior vena cava through the vertebral vessels [7].

Ten patients had another thyroid pathology, although those primary pathologies like papillary carcinoma, hypothyroidism, or goiter are not rare. On the other hand, the blood distribution to the thyroid is rich, with about 3-4% of total cardiac output supplying the thyroid gland. The iodine-rich and hyperoxic environment in some of these primary thyroid pathologies might inhibit the development of metastatic cells by physiological and chemical mechanisms, respectively, as was suggested by Willis [4].

Although colon metastasis to the thyroid represents an advanced stage of disease and is usually associated with lung and liver metastasis, attempts should be made to reach a point where the patient can be reasonably verified with no further evidence of metastasis. Once metastasis to the thyroid is suspected by imaging, complete thyroidectomy should be performed because the likelihood of colon metastasis to thyroid in the presence of other organ involvement has been seen to be higher than that in primary thyroid neoplasia. 

## Figures and Tables

**Table 1:** 

Case number	Paper	Age/gender	Location	Staging (Duke)	Adjuvant therapy	Time to occurrence of thyroid metastasis (years)	Associated thyroid pathology or neoplasia	Other organs involvement	Treatment	Prognosis
1	Willis 1931 [4]	54 M	Right colon	III	No	4	No	Liver, kidneys, lungs	No	—

2	Sklaroof, 1954	73 F	Rectum	—	—	7	Colloid nodule	No	Prophylactic tracheostomy	two months after diagnosis while attempting tracheostomy.

3	Elliott and Frantz, 1960 [1]	56 F	Rectum	—	—	Synchronous	Hashimoto's thyroiditis	—	Total thyroidectomy and RND	3 months

4	Shimaoka et al. 1962 [2]	50 F	Sigmoid colon	—	—	4	No	Lungs	Complete thyroidectomy	—

5	Wychulis et al., 1964 [3]	37 F	Rectum	III	No	0.5	No	Lungs	Rt thyroidectomy and isthmectomy	3 months

6	B. Make et al., 1974	68 M	Sigmoid colon	—	—	4	—	Liver	Radiation	8 months

7	J. A. Thomson 1975	44 F	Right colon	—	5fu	4	No	Brain	Thyroidectomy	9 months since clinical signs

8	T. Ishida et al., 1982	34 M	Rectum	II	Chemo and immunotherapy for 10 months	2	—	Generalized metastasis	Total thyroidectomy	7 months

9	J. W. Lester Jr. et al., 1986	56 F	Colon	—	—	2.5	—	Liver, Lungs	—	—

10	C. Rigaud et al., 1987	68 F	Colon	—	Yes (no spec)	2	—	Yes	Thyroidectomy and chemotherapy	3 months

11	C. Rigaud et al., 1987	77 M	Rectum	—	Yes (no spec)	4	—	Yes	Thyroidectomy and chemotherapy	1 month

12	E. G. Cristallini et al., 1990	64 F	Colon	—	—	4	No	Liver	Thyroidectomy	>12 months

13	D. Nachtigal et al., 1992	69 F	Left colon	II	No	8	No	Lungs	Total thyroidectomy	8 months

14	Y. Shibutani et al., 1992	52 F	Sigmoid colon	IV	—	3	No	Lungs	Subtotal thyroidectomy	8 months

15	S. Kim et al. 1994	37 F	Colon	—	—	7	—	Lymph nodes, skin	—	>2 months

16	T. W. Mesko et al., 1996	59 F	Rectum	II	5fu, leucovorin, levamisole, rx.	2	No	Vertebrae, kidney	Right thyroidectomy and isthmectomy. Radiation to vertebrae	>36 months

17	P. Osin et al., 1996	70 F	Rectosigmoid colon	IV	—	Synchronous	Thyroid colloid nodule	Liver	Rt thyroid lobectomy and isthmectomy	—

18	S. Takashima et al., 1998	67 M	Rectum	—	—	2	Hashimoto's	—	—	—

19 table	C. H. Kim et al., 1999	68 F	Sigmoid colon	II	No	2	No	Lungs	Left lobe thyroidectomy, radiation	—

20	K. Kameyama et al., 2000	82 M	Sigmoid colon	III	—	2	Follicular adenoma	Peritoneum, liver, lungs, kidneys, bones.	No	Died of MOF 2 years after colectomy

21	M. De Ridder et al., 2001	75 F	Left colon	III	—	7	Hyperthyroidism	No	Total thyroidectomy radiation	Disease recurs 4 months later. Pts died 9 months after the thyroid recurrence

22	M. S. Boleas Aguirre et al., 2001	80 F	Colon	—	—	7	—	Larynx	—	—

23	K. Akimaru et al., 2002	67 M	Right colon	III	—	6	No	Brain, lungs	Chemotherapy	4 months

24	G. P. Perinu et al., 2003	43 M	Left colon	—	—	4	MNG	Liver	—	—

25	R. L. Witt, 2003	71 M	Colon	III	—	7	Hurthle cell neoplasia	Lungs, liver	Rt thyroid lobectomy and isthmectomy	—

26	Fujita et al., 2004 [5]	28 F	Rectum	IV	5FU, CISPLATIN	Synchronous	No	Lungs	Left thyroid lobectomy and modified neck dissection	6 months

27	O. Fadare et al., 2005	59 F	Sigmoid colon	IV	—	Synchronous (incidental finding)	Follicular neoplasia	Liver, peritoneum	Thyroidectomy, chemotherapy	>12 months

28 table	Phillips et al. 2005 [6]	81 F	Colon	III	—	2	No	No	Total thyroidectomy and laryngectomy	—

29	J. C. Youn et al., 2006	85 M	Right colon	IIIB	5FU, LEUKOVORIN	1.5	Hypothyroidism	—	Irinotecan and fluoropyrimidine	—

30	K. Kumamoto et al., 2006	66 F	Right colon	I-II	5FU, Leucovorin	3.5	hypothyroidism	Liver, lungs	Left lobe thyroidectomy and cervical lymph nodes dissection	—

31	W. C. Hanna et al., 2006	48 F	Distal colon	IV	Yes	Synchronous	No	Lungs	Rt thyroid lobectomy and isthmectomy	—

32	I. Cozzolino et al., 2010	60 M	Rectosigmoid colon	IV	FOLFOX-4 (8 cycles) After lung met FOLFIRI, FOLFIRI + cetuximab. Mitomycin C + capecitabine, zoledronic acid RX	6	None	Lungs, liver, bones	Radiation	>72 months

33 abs	K. Nakamura et al., 2011	58 F	Sigmoid colon	—	—	5	None	Lung	Subtotal thyroidectomy, FOLFOX and bevacizumab	—

34	Goatman et al. 2012	82 M	Rectum and cecum	—	Refused	5	Papillary	Lung	Thyroidectomy, left neck dissection	—

35	Our case	62 F	Sigmoid colon	III	5 FU, leucovorin, oxaliplatin	5	None	Lung	Thyroidectomy, lung lobectomy, and re-colectomy	>24 months
